# Calibrating coseismic coastal land-level changes during the 2014 Iquique (*M_w_*=8.2) earthquake (northern Chile) with leveling, GPS and intertidal biota

**DOI:** 10.1371/journal.pone.0174348

**Published:** 2017-03-23

**Authors:** Eduardo Jaramillo, Daniel Melnick, Juan Carlos Baez, Henry Montecino, Nelson A. Lagos, Emilio Acuña, Mario Manzano, Patricio A. Camus

**Affiliations:** 1 Instituto de Ciencias de la Tierra (ICT), Facultad de Ciencias and TAQUACH (Transdisciplinary Center for Quaternary Research), Universidad Austral de Chile, Valdivia, Chile; 2 Centro Sismológico Nacional, Universidad de Chile, Santiago, Chile; 3 Departamento de Ciencias Geodésicas, Universidad de Concepción, Los Angeles, Chile; 4 Centro de Investigación e Innovación para el Cambio Climático (CIICC-UST), Facultad de Ciencias, Universidad Santo Tomás, Santiago, Chile; 5 Programa de Doctorado en Biología Marina, Universidad Austral de Chile, Valdivia, Chile; 6 Centro de Investigación en Biodiversidad y Ambientes Sustentables (CIBAS), Departamento de Ecología, Facultad de Ciencias, Universidad Católica de la Santísima Concepción, Concepción, Chile; Universita degli Studi di Genova, ITALY

## Abstract

The April 1^st^ 2014 Iquique earthquake (*M*_*W*_ 8.1) occurred along the northern Chile margin where the Nazca plate is subducted below the South American continent. The last great megathrust earthquake here, in 1877 of *M*_*w*_ ~8.8 opened a seismic gap, which was only partly closed by the 2014 earthquake. Prior to the earthquake in 2013, and shortly after it we compared data from leveled benchmarks, deployed campaign GPS instruments, continuous GPS stations and estimated sea levels using the upper vertical level of rocky shore benthic organisms including algae, barnacles, and mussels. Land-level changes estimated from mean elevations of benchmarks indicate subsidence along a ~100-km stretch of coast, ranging from 3 to 9 cm at Corazones (18°30’S) to between 30 and 50 cm at Pisagua (19°30’S). About 15 cm of uplift was measured along the southern part of the rupture at Chanabaya (20°50’S). Land-level changes obtained from benchmarks and campaign GPS were similar at most sites (mean difference 3.7±3.2 cm). Higher differences however, were found between benchmarks and continuous GPS (mean difference 8.5±3.6 cm), possibly because sites were not collocated and separated by several kilometers. Subsidence estimated from the upper limits of intertidal fauna at Pisagua ranged between 40 to 60 cm, in general agreement with benchmarks and GPS. At Chanavaya, the magnitude and sense of displacement of the upper marine limit was variable across species, possibly due to species—dependent differences in ecology. Among the studied species, measurements on lithothamnioid calcareous algae most closely matched those made with benchmarks and GPS. When properly calibrated, rocky shore benthic species may be used to accurately measure land-level changes along coasts affected by subduction earthquakes. Our calibration of those methods will improve their accuracy when applied to coasts lacking pre-earthquake data and in estimating deformation during pre–instrumental earthquakes.

## Introduction

Rapid large-scale, natural disturbances are a key factor in shaping the environment (e.g. [1–4]) and are thus an integral part of plant and animal communities around the world. Volcanic eruptions, earthquakes, tsunamis, floods and fires leave lasting ecological effects [5–8]; such disturbances may therefore affect growth, reproduction and mortality of organisms [9] as well as spatial and temporal patterns of ecosystems across landscapes (e.g. [10,11,12]).

Understanding the consequences of natural disturbances is critical, not only to inform resource managers about present-day natural communities, but also to predict the effects of future disturbances and their consequences on the environment. Understanding disturbances requires study of damage, patterns of recolonization, resilience and reaccommodation of affected organisms and communities [13]; however, these methods are preconditioned by the availability of data before and after the disturbance. Before—after comparisons of the effects of a large storm in England [14] and those associated with the 2010 and 2011 earthquakes in Chile and Japan (*M*_w_ 8.8 and 9,1, respectively) [8, 11], are examples of such a serendipitous availability of data used to evaluate effects. During the storm in October 1987, strong winds demolished millions of trees in southeast England [14, 15]. Due to a prior national ecological survey along 103 woodlands in that region, ecological data collected in 1971 and 2002 could be compared; the comparisons showed an increase in understory plant species richness in areas exposed to the storm [14]. Intertidal surveys carried out shortly before and immediately after the Maule earthquake, showed that along the co-seismically uplifted Arauco Peninsula (ca. 37°S), sandy beach habitats widened and flattened. Upper and mid intertidal habitats were restored seaward of coastal armoring and were rapidly colonized by mobile crustaceans who had been formerly excluded due to the presence of seawalls and rocky revetments [11]. In turn, along the coastline affected by subsidence during the 2011 Tohoku earthquake, the comparison of before and after data together with a continuous monitoring showed a slow recovery of the sandy beaches and their habitats [8].

The Chilean coast runs along the subduction zone formed by convergence of the Nazca oceanic plate beneath the South American continent that commonly generates great megathrust earthquakes and accompanying tsunamis [15–18]. Plate convergence at ~ 66 mm/yr results in great (*M*_*w*_>8.5) earthquakes on average every 150–200 years [19], which rupture the shallower part of the plate boundary located mostly offshore. Because of the offshore location of these great earthquakes, the mainland coasts is mostly affected by subsidence, whereas localized uplift may occur on peninsulas and islands located closer to the subduction trench [17,20]. The mainland coast may also emerge as a result of modest (*M*_*w*_ 7–8) earthquakes that occur at greater depths (> 35 km) reaching below the coastline [21], such as the 1995 Antofagasta (*M*_*w*_ 8), 2007 Tocopilla (*M*_*w*_ 7.7) and 2012 Constitución (*M*_*w*_ 7) earthquakes.

The northern segment of the subduction plate boundary, nearly 500 km [19,22] between 24 and 17°S last ruptured in the great (*M*_*w*_ >8.8) earthquake of 1877 and triggered a transpacific tsunami with causalities in Hawaii and Japan [16]. Given that no major earthquakes had affected this region since 1877, it had long been considered as one of the most mature seismic gaps in South America, capable of generating a *M*_*w*_ >8.8–9 earthquake. The 2014 Iquique earthquake filled only a fraction of this seismic gap, leaving a ~300-km-long unruptured segment capable of producing an *M*_*w*_ ~8.5 event [23–25] located between Iquique and Mejillones. A rapid assessment of the rupture length along coasts affected by such megathrust earthquakes is important, not only for estimating damages to local infrastructure and loss of economic resources, but also to estimate the extent of plate-boundary slip and evaluate the probability for future earthquakes occurring on neighboring segments. Such a rapid assessment may also provide insight on the possible triggering of secondary structures in the upper plate, such as the Pichilemu Fault that generated a *M*_*w*_ 6.9 earthquake, 11 days after the 2010 Maule earthquake [26]. Space geodesy provides the most accurate method together with the analysis of seismic waves to estimate the rupture length and moment magnitude of great subduction earthquakes. However, because Global Positioning System (GPS) stations are usually distributed over broad regions at tens, to sometimes hundreds, of kilometers spacing they provide only a rough estimate of the rupture length and a smoothed distribution of the plate-boundary slip. Radar interferometry, in turn, requires repeated satellite observations that are usually weeks apart, and thus may hide differences in deformation induced by the main shock and following aftershocks as well as by fault after slip [27]. The integration of GPS, InSAR, and resurveyed benchmarks with elevation measurements of displaced rocky intertidal fauna provides an unusually accurate robust assessment of local deformation during great earthquakes [28].

The intertidal biota of rocky shores along the world coastlines, are characteristically zoned in biotic belts [29–31] usually referenced to specific tidal levels such as high, middle and low tidal zones (e.g. [30–32]) Littorinid gastropods (Mollusca) and barnacles (Crustacea, Cirripedia) characterize the higher zones of rocky shores around the world, whereas mussels (Mollusca, Bivalvia), brown and coralline algae are typical components of the middle and lower zones at those shores [29–31, 33]. Due to their distinct vertical zonation, some of these organisms have been used to measure coastal vertical displacements during great earthquakes [17,20,34,35]. Fitzroy and Darwin [36] pioneered the use of intertidal rocky shore invertebrates to estimate coseismic land-level changes caused by the great 1835 (M>8.5) earthquake in south-central Chile [37].

**Fig 1:**
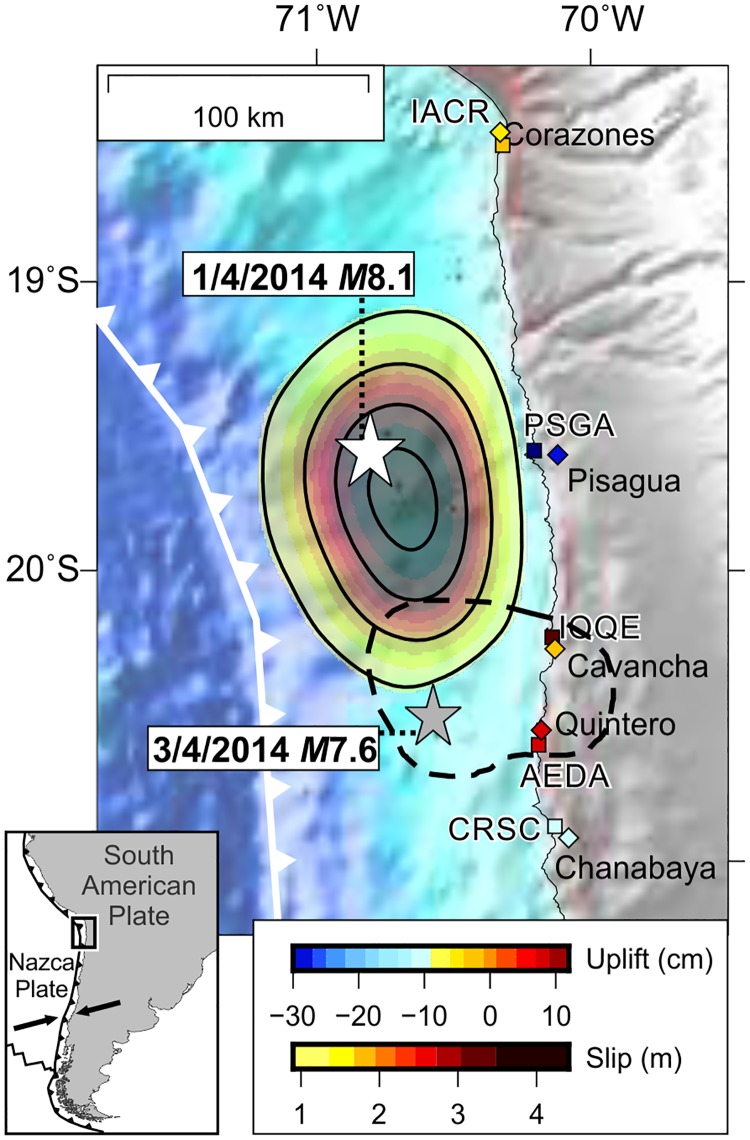
Tectonic setting, observed land-level changes and plate-boundary slip during the Iquique earthquake. Stars indicate epicentres of the mainshock and major aftershock [25]. Squares denote location of the benchmarks, campaign GPS and intertidal biota sites studied, while diamonds indicate location of continuous GPS stations used in this study (S1 Table). Color-coded by static uplift (see text for details). Arrows in inset show plate convergence at ~ 66 mm/yr.

Coastal land-level changes estimated from rocky-shore biota have been measured after several great earthquakes and used to provide a rapid assessment of the rupture length [11,17,26,34,38,39]. The accuracy of such markers is influenced by various factors, which are in many cases site dependent and thus difficult to assess without previous calibration studies. Here we present a calibration of land-level changes estimated from benchmarks surveyed both with tidal levels and GPS as well as from variability in the upper level of the vertical distribution of various intertidal organisms, shortly before and after the 2014 Iquique event (Fig 1).

We started surveying the coast of northern Chile eight months before the 2014 Iquique earthquake, and conducted the first post-earthquake survey ten days after to measure land-level changes. We hypothesize that such land-level changes are the most important exogenous disturbance to the ecology of sessile benthic invertebrates and macroalgae as measured by changes in vertical distribution (zonation). We tested this hypothesis by comparing: i) pre- and post- earthquake measurements collected at stationary benchmarks installed on upper shore levels, and ii) upper levels of vertical distribution of intertidal rocky shore biota. We compare these different types of measurements among sites along the Iquique rupture zone and beyond (Fig 1) and propose a simple methodology to quantify vertical coseismic displacements during earthquakes. We discuss the accuracy of each method based on a comparison with resurveyed campaign GPS benchmarks installed in our rocky shore sites as well as with nearby continuous GPS stations.

### Coastal biogeographic setting and rocky shore species

The study sites (Fig 1) are located within the “Norte Grande” (Big North; 18–27°S), a biogeographically complex area encompassing the southern half of the Warm-Temperate Peruvian Province (∼4–33°S; see review by Thiel *et al*. [40]), where warm-water species are virtually absent despite the coast was repeatedly invaded by tropical taxa through the Quaternary [41–43]. Such features started to form during the late Paleogene, when the onset of the cold Humboldt Current system, and the subsequent onset of its oxygen minimum zone, led to the northward migration of the former warm biota and to mass faunal extinctions [40,44–46]. The impact of past time events is thus clearly signaled in the spatial genetic structure of many common taxa along the Big North (see [40,47] and references therein). However, modern patterns of species distribution are largely driven by oceanographic, climatic and topographic factors determining patterns of coastal upwelling, larval transport and primary productivity, either on mesoscales or on large scales in connection with remote equatorial processes associated with El Niño events (see [40,48–52]).

Big North coastal communities remain poorly known compared to those south of 27°S [40]. Rarity and high spatial turnover are prevailing traits on rocky intertidal shores, where most species (>50%) occur in few localities (∼30%), producing a high between-site variation in community richness, composition and evenness [53]. These communities show a high temporal turnover, and species composition may change up to 40–50% between consecutive seasons [52]. Big North rocky shore intertidal communities experience the strongest and most direct impacts of the El Niño-Southern Oscillation (ENSO) (e.g., [40,50,51]), which may partly explain differences in intertidal zonation and dominance patterns observed on small spatial scales [51]. However, a small group of core species with the highest abundance and widest distribution in this region are the most representative of different intertidal zones on rocky shores [40,53,54]: (a) a higher zone dominated by the periwinkle *Echinolittorina peruviana* and the barnacle *Jehlius cirratus*; (b) a middle zone where mussels (*Perumytilus purpuratus)*, barnacles (*Notochthamalus scabrosus)* and leshy-encrusting and frondose algae such as the red algae *Hildenbrandia lecanellieri*, the green algae *Ulva rigida* and *Pseudoulvella* sp., and the brown algae *Colpomenia* spp. and *Ralfsia* spp., are the most common taxa; (c) a lower zone dominated by the brown kelp *Lessonia berteroana*, and a guild of encrusting coralline algae (mainly the genera *Lithophyllum*, *Phymatolithon*, *Spongites* and *Titanoderma*) and erect coralline algae (*Corallina officinalis*).

## Methods

No specific permits were required for the described intertidal field studies. The rocky shores we studied in Chile are unrestricted to public access and use, and are not privately owned or designated as protected areas (reserves or parks). No protected or endangered species were involved in this study.

### Variability of land-level change measurements

Benchmarks consisting of 4 cm length stainless steel bolts were installed in August 2013 on the upper rocky shore levels at six sites (Fig 1 and S1 Table). We measured the elevation between the benchmarks and the tide, and repeated the measurements after the earthquake (Table 1). Benchmark heights were referred to mean tide—level datum using the TPXO v8 Atlas model [55]. TPXO is a predictive model based on harmonic constituents extracted from >12 years of TOPEX/POSEIDON satellite altimetry data. The model has been used for similar studies of land-level changes associated with earthquakes in south-central Chile [28] and Sumatra [56] and validated with tide gauge data in south-central Chile [28]. A Trimble GPS receiver was used to survey points near benchmarks during between 2 and 3 hours in August 2013 and May 2014 at five sites (Table 1). The data collected at Chanavayita were used for comparisons with the leveling data collected at Chanavaya (*ca*. 20 km south).

**Table 1:** Times of data collection at the sites where benchmarks (X) were measured and GPS mobile stations were installed for field campaigns (●).

	pre—earthquake	post—earthquake			
sites	August 2013	April 2014	May 2014	June 2014	August 2014
Corazones	X			X	X
Pisagua norte	X ●	X	X ●	X	X
Pisagua sur	X ●	X	X ●	X	X
Cavancha	X ●	X	X ●		
Quintero	X ●	X	X ●		X
Chanavayita	●		●		
Chanavaya	X	X	X		

Daily solutions of additional five continuous GPS stations were processed, using the same constraints and used to estimate corresponding land-level changes at selected observation periods coincident with the benchmark leveling and campaign GPS. The processing strategy included a dense network of reference stations located in the southern part of South America and processed with Bernese 5.0 Software [57–59].

A week of daily positions estimated from the continuous GPS stations were averaged during each post-earthquake period when intertidal biota was surveyed, and compared with the corresponding pre-earthquake observation period to estimate land-level changes and make direct comparisons of data collected by campaign and continuous GPS. By using this approach instead of measuring only the static displacement induced by the earthquake, we take into account the vertical components induced by the seasonal hydrological cycle, transient post-seismic movement such as after slip and viscoelastic mantle rebound [60], and other sources of uncertainty. We also computed trajectory models of the GPS daily position time series using the method of Bevis and Brown [61], including jumps caused by antenna changes and local earthquakes from the NEIC Catalogue (located at *d* ≤ 10^(0.5 * *mag*– 0.8)^, where *d* is the distance between the epicentre and the GPS station and *mag* is the earthquake moment magnitude), annual and semi-annual seasonal periodic variations, the secular pre-earthquake linear inter seismic trend, and a transient logarithmic post-seismic trend (see [21,61] for details).

### Pre—earthquake and post—earthquake zonation of the rocky shore biota

The elevation of the upper limits of selected rocky shore biota with respect to tide levels were measured at Pisagua Norte, Pisagua Sur and Chanavaya. The selected sessile biota included the barnacle *Jehlius cirratus*, the mussel *Perumytilus purpuratus*, the seaweed *Lessonia berteroana* and two calcareous algae (an erect form (*Corallina officinalis*)) and an encrusting calcareous lithothamnioid algae). Differences in height between the upper limits of rocky shore biota and low tide were corrected as explained above.

### Statistical analysis

The direction and amount of the vertical displacement of the rocky intertidal zones at each site were compared by assessing the differences in elevation above mean tide level (H) of benchmarks and the upper limits of the vertical distributions of the five selected sessile species. For the benchmarks only a single measurement of elevation was available for the pre-earthquake period (August 2013). We used a two-tailed, one-sample *t*-test to compare the averaged H of the replicated samples collected during each of the post-earthquake sampling periods with the un-replicated pre-earthquake data. For the upper limits of the sessile species, we used two observed distributions with mean H_1_ and H_2_ for upper vertical limits recorded during pre- and post-earthquake periods, respectively. Because only a limited number of measurements could be collected during the post–earthquake period, we pooled the data from Pisagua Norte and Pisagua Sur, as well as those from Chanavaya.

The averaged differences in elevations between sampling dates were calculated as: ΔH = H_2_—H_1_, with negative and positive values of ΔH indicating subsidence and uplift, respectively. To avoid the non-independence of all pair-wise differences between H_1_ and H_2_, we approximated the distribution of ΔH through simulations in a Bayesian framework [62]. Using a uniform non-informative prior distribution, we simulated 1000 random values from each normal distribution of H using the observed means of H_1_ and H_2_ and their standard deviations S_1_ and S_2_. We computed the difference between H_1_ and H_2_ for each pair of simulated and independent values, and estimated the 95% Student’s *t—*test confidence interval for the posterior distribution of ΔH. This analysis is supported by the observation that the distribution of intertidal biota elevations of sessile species is usually not significantly different from Gaussian [28].

## Results and discussion

Detailed observations on vertical distributions of rocky shore organisms may be used to define mean tide level datum and measure sudden changes in relative sea level, caused by coastal deformation during earthquakes [63]. However, in order to use such biotic makers of past sea-level positions to accurately estimate coseismic land-level changes associated with a seismic event, calibration studies are necessary for each species to validate the results against independent geodetic methods.

Coseismic coastal land-level changes estimated from averaged mean elevations of benchmarks surveyed before and after the 2014 Iquique earthquake in northern Chile, are shown in Table 2. Negative values indicate subsidence for Corazones (~ 3–9 cm), Pisagua Norte and Pisagua Sur (~ 30–50 cm) and uplift of ~15 cm at Chanabaya. The uplift is associated with the *M*_*w*_ 7.6 aftershock that occurred 2 days after the mainshock at greater depths reaching below the coastline (Fig 1). On the other hand, positive and negative values estimated at Quintero (Table 2) suggest that this site is located near the hinge line that separates sectors of coseismic subsidence and uplift. Identifying the precise location of the hinge line is important for constraining the slip earthquake distribution accurately [58].

**Table 2:** Pre—earthquake and post—earthquake elevations levels (cm) of benchmarks along rocky shores of northern Chile. Statistical differences were assessed by using a one sample t—test, considering the value (H1, n1 = 1) recorded during the pre—earthquake period as a constant, compared to data recorded during the post—earthquake period (H2; n2).

	pre—earthquake (August 2013)		post—earthquake (April—August 2014)					one sample *t*—test		
sites	H_1_	n_1_	months	H_2_	n_2_	SD	95% CI	H_2_—H_1_	*t*—statistic	P—value
Corazones	267.5	1	June	264.8	10	5.14	(261.15, 268.51)	-2.7	-1.64	0.135
			August	258.1	20	6.09	(255.29, 260.99)	-9.4	-6.88	**0.000**
Pisagua norte	289.5	1	April	253.0	10	8.24	(247.05, 258.85)	-36.6	-14.03	**0.000**
			May	253.3	10	3.01	(251.12, 255.49)	-36.2	-37.47	**0.000**
			June	261.0	10	1.76	(259.74, 262.26)	-28.5	-51.24	**0.000**
			August	239.6	6	2.38	(237.12, 242.12)	-49.9	-51.26	**0.000**
Pisagua sur	241.2	1	April	199.4	10	8.99	(192.95, 205.81)	-41.8	-14.71	**0.000**
			May	203.7	10	3.62	(201.09, 206.27)	-37.5	-32.78	**0.000**
			June	202.9	10	2.04	(201.43, 204.35)	-38.3	-59.37	**0.000**
			August	201.8	4	7.67	(189.59, 214.01)	-39.4	-10.27	**0.002**
Cavancha	174.2	1	April	180.6	10	7.37	(175.28, 185.82)	6.4	2.72	**0.023**
			May	161.8	5	2.15	(159.10, 164.43)	-12.4	-12.96	**0.000**
Quintero	261.5	1	April	253.3	10	1.56	(252.20, 254.42)	-8.2	-16.65	**0.000**
			May	266.6	10	8.79	(260.27, 272.85)	5.1	1.82	0.102
			August	270.0	10	1.95	(268.65, 271.43)	8.5	13.86	**0.000**
Chanavaya	270.3	1	April	287.3	10	3.57	(284.76, 289.86)	17.0	15.08	**0.000**
			May	283.8	10	7.29	(278.60, 289.04)	13.5	5.86	**0.000**

**Fig 2:**
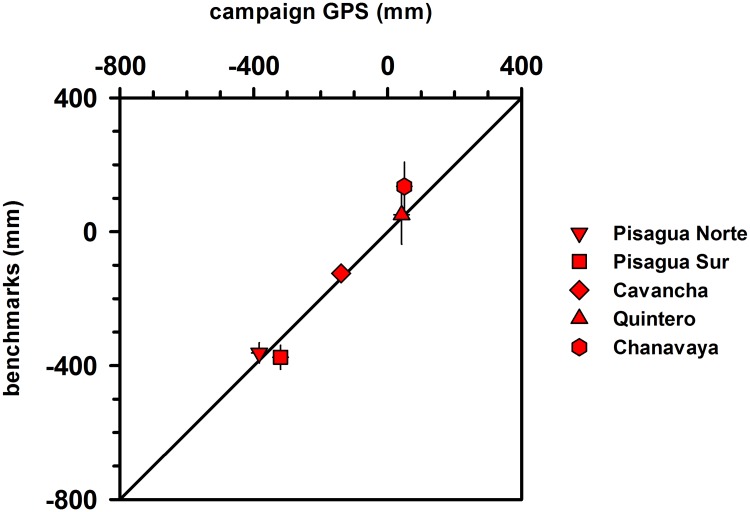
Comparison of land level changes estimated during May 2014 using measurements of benchmarks with respect to surveyed tidal datums and campaign GPS.

**Fig 3:**
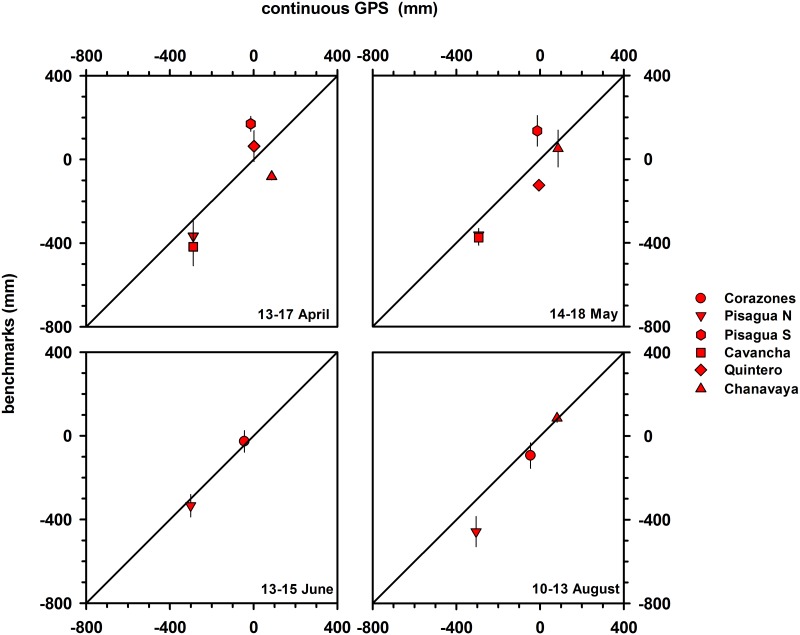
Comparison of land level changes estimated during April, May, June and August 2014, using surveyed benchmarks and continuous GPS stations. Averaged weekly positions were estimated from daily solutions of the continuous GPS data for benchmark surveying periods.

Although similar estimates of land-level changes were obtained from benchmarks and campaign GPS data at Pisagua Norte, Pisagua Sur, Cavancha and Quintero, these two methodologies yielded different results at Chanavaya (Table 3 and Fig 2). The discrepancy may be related to the fact that the campaign GPS used for the comparisons was located 20 km north of Chanavaya, and thus the spatial variability in coastal deformation that commonly characterizes great earthquakes over such distances could not be estimated [28]. Land-level changes estimated from benchmarks are closer to campaign GPS measurements than they are to continuous GPS measurements (Table 3 and Fig 3). This difference in precision is likely also a result of the spatial distances over which the comparisons between benchmarks and continuous GPS measurements were made.

**Table 3:** Pre—earthquake to post—earthquake differences in shore elevation measured by levelling (elevation of benchmarks), campaign and continuous GPS stations. Values in parentheses are standard deviations for benchmarks and standard errors for the data of campaign and continuous GPS.

		pre—earthquake and post—earthquake (cm)		
sites	dates	benchmarks	campaign GPS	continuous GPS
Corazones	June 14—August 13	-2.7 (5.1)		-4,4 (0.1)
	August 14—August 13	-9.4 (5.9)		-4.6 (0.4)
Pisagua Norte	April 14—August 13	-36.6 (8.2)		-28.9 (0.6)
	May 14—August 13	-36.2 (3.1)	-38,4 (2,3)	-29.3 (0.4)
	June 14—August 13	-28.5 (1.8)		-30.1 (0.5)
	August 14—August 13	-49.9 (2.4)		-30.6 (0.5)
Pisagua Sur	April 14—August 13	-41.8 (9.0)		-28.9 (0.6)
	May 14—August 13	-37.5 (3.6)	-32.0 (2.3)	-29.3 (0.4)
	June 14—August 13	-38.3 (2.0)		-30.1 (0.5)
	August 14—August 13	-39.4 (7.7)		-30.6 (0.5)
Cavancha	April 14—August 13	6.4 (7.4)		0.2 (0.4)
	May 14—August 13	-12.4 (2.1)	-13.9 (2.3)	-0.5 (0.2)
Quintero	April 14—August 13	-8.2 (1.6)		8.6 (0.6)
	May 14—August 13	5.1 (8.8)	4.2 (2.3)	8.6 (0.5)
	August 14—August 13	8.5 (1.9)		8.1 (0.5)
Chanavaya	April 14—August 13	17.0 (3.6)		-1.4 (0.4)
	May 14—August 13	13.5 (7.3)	5.0 (2.3)	-1.3 (0.3)

**Fig 4:**
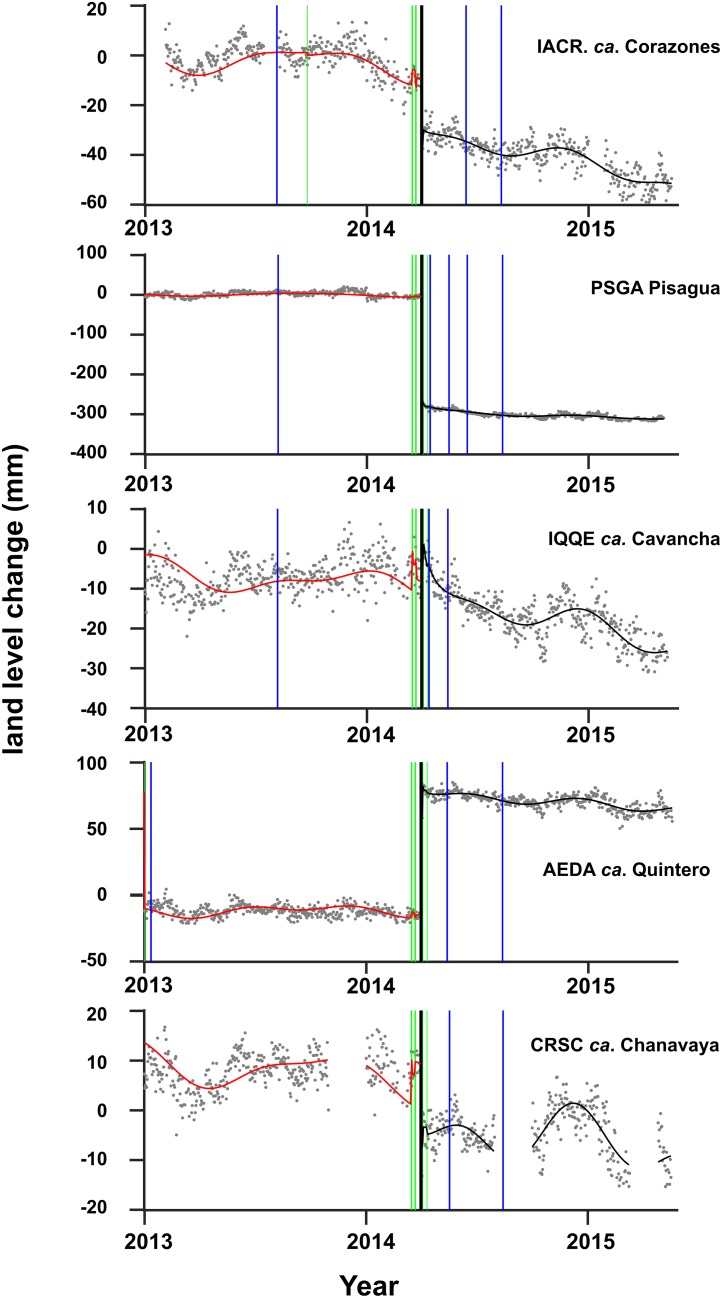
Time series of land-level changes estimated from daily positions of continuous GPS used in this study. The thick black line marks the time of the 2014 Pisagua earthquake. The red and black lines show trajectory models calculated to separate epochs before and after the earthquake. Green lines mark Heaviside jumps either caused by local earthquakes or antenna changes. Blue lines mark the dates when benchmarks and rocky shore biota were surveyed. See Fig 1 for location of the sites.

The five continuous GPS stations show consistent land-level changes during the 2014 earthquake sequences (Fig 4). However, three stations (IACR, IQQE, CRSC) show scatter in the daily positions, which likely result from a combination of monument instability, weak substratum, and multipath as well as other site-specific processes difficult to assess. These three stations are also significantly influenced by seasonal variations mostly resulting from the local hydrological cycle and the elastic response of the solid Earth to changes in loading from surface and groundwater flow. This effect reaches maximum amplitude of ~2 cm at CRSC. In turn, stations PSGA and AEDA show very consistent positions throughout the years that preceded and followed the 2014 earthquake. Interestingly, these stations show the opposite sense of displacement during the earthquake sequence.

A detailed inspection of the time series revealed that AEDA was uplifted ~8 cm during the Mw 7.6 aftershock that occurred on April 3^rd^ and affected only the southern part of the main shock rupture zone (Fig 1). This aftershock was a typical domain-C [64] event that ruptured deeper than the main shock at the Moho-slab boundary; such events have been associated with the rise of the central Andean coastline [21]. In turn, PSGA shows ~30 cm of subsidence associated with the mainshock that occurred directly offshore adjacent to the coast in the shallower domain B. IQQE is located at the limit of the main- and aftershock rupture zones, and therefore shows a combination of minor subsidence followed by a few mm of uplift, over imposed to significant scatter that reaches an amplitude of more than a cm. This might be an effect of the urban location of the station adjacent to the city of Iquique. The analysis of these continuous GPS stations shows that even if these are considered state-of-the-art geodetic instruments, the estimates of land-level changes may be site dependent and contain uncertainties of several cm associated to site-specific processes that are difficult to evaluate and quantify, before analyzing several years of data.

Coastal subsidence caused the upper limits of the vertical distribution of the whole species pool to decrease significantly at Pisagua, which we estimate at values ranging—26.0 to nearly—60.0 cm using the lithothamnioid calcareous algae and the mussel *Perumytilus purpuratus*, respectively (Table 4). For Chanavaya, the magnitude and sense of displacement of the upper vertical limit was variable across species: there was no significant change estimated from the barnacle *Jehlius cirratus*, whereas we measured ~ 11 cm of subsidence using the brown algae *Lessonia berteroana* (Table 4). The use of the mussel *Perumytilus purpuratus* and the calcareous algae *Corallina officinalis* and the Litothamnioid calcareous taxon yielded coastal uplift varying from ~ 9 to 21 cm (Table 4).

**Table 4:** Pre–earthquake and post-earthquake upper limits (cm) for sessile rocky shore species and estimated change (ΔH) in elevation between pre—earthquake and post—earthquake periods at Pisagua and Chanavaya. Each ΔH estimate was based on 1000 independent simulated values. M = mean, SD = standard deviation.

	pre—earthquake (H_1_)			post—earthquake (H_2_)			simulated differences in elevations (cm)			
sites and species	M	SD	n	M	SD	n	ΔH	95% CI	T	P
Pisagua										
*Jehlius cirratus*	122.1	3.7	2	69.5	9.5	9	- 51.9	- 48.0, - 55.7	- 26.35	< 0.001
*Perumytilus purpuratus*	107.1	6.2	2	52.2	16.6	9	- 59.9	- 53.8, - 66.1	- 19.19	< 0.001
*Lessonia berteroana*	50.1	17.5	2	2.2	8.4	9	- 58.2	- 28.5, - 87.9	- 3.85	< 0.001
*Corallina officinalis*	53.6	16.8	2	- 17.2	20.6	2	- 53.6	- 18.6, - 88.6	- 3.01	0.003
Lithothamnioid calcareous algae	21.6	11.5	2	- 26.0	9.1	7	- 40.1	- 28.0, - 52.2	- 6.52	< 0.001
Chanavaya										
*Jehlius cirratus*	208.8	19.1	2	195.4	22.3	4	- 1.2	- 11.9, 9.5	- 0.21	0.832
*Perumytilus purpuratus*	138.8	9.2	2	146.7	11.7	4	9.2	2.8, 15.6	2.82	0.005
*Lessonia berteroana*	19.8	0.7	2	8.4	20.1	4	- 10.7	- 11.8, - 9.7	- 20.83	< 0.001
*Corallina officinalis*	13.8	2.1	2	25.4	25.1	4	11.8	7.3, 16.3	5.17	< 0.001
Lithothamnioid calcareous algae	- 14.7	11.3	2	6.2	18.4	4	20.8	19.9, 21.6	46.0	< 0.001

Physical factors and ecological processes operating at different spatial or temporal scales probably affect these differences and the accuracy of our estimates (e.g., tidal height, desiccation, thermal stress, foraging patterns and biological interactions [52, 65, 66, 67, 68]). For instance, high tidal distribution of rocky shore organisms is closely related to air exposure and dessication (e.g. [65]). It has been found that macroalgae, barnacles and mussels had higher upper limits along the exposed coasts of Islas Cies (España), as compared with sheltered locations (e.g. [65]). In northern Chile, barnacles (i.e., *Jehlius cirratus* and *Notochthamalus scabrosus*) show a persistent recruitment pattern, characterized by a positive relationship between spatial occurrence and persistence along time, which is highly correlated from one coastal segment to another from a few to tens of kilometers along the Chilean coast [66]. In particular, at Chanavayita and nearby sites in northern Chile, *Jehlius cirratus* has shown consistently high recruitment rates across the upper tidal level, leading to persistent patterns of fast recolonization of vacant space at monthly intervals [67]. These spatially persistent recolonization patterns, suggest that the maintenance of the upper intertidal limits of barnacles can be a reliable measurement for coseismic coastal deformation. Moreover, the position and maintenance of the upper limits of intertidal macroalgae are influenced by physical and biological processes [51, 68]. In northern Chile, the abundance and upper shore vertical limits of *Lessonia berteroana* may exhibit between-site variations related to differential impacts of El Niño events [51], and experimental studies at Punta Patache (south of our site located at Chanavayita) showed that the upper limit of this macroalgae is modulated by grazing effects [68].

Our integrated analysis shows that in general, rocky shore sessile species can provide accurate measurements of land-level changes during earthquakes. Due to time constraints we chose to survey different species and measure tide levels for positioning the benchmarks with respect to mean tide and deploying the campaign GPS stations over making a greater number of measurements. Our study highlights the need to conduct regular surveys of coastal zones prone to large earthquakes in order to determine the local variability of species-specific patterns of vertical distribution, and thus determining which may be most accurate for assess land-level changes. s. The choice of species will be largely dependent on the expected amplitude of the coseismic land-level changes [11, 69]; for example, in areas such as the Arauco Peninsula where slip occurred directly below the coast resulting in meters of coastal uplift, most species would provide relatively accurate results; in contrast, regions affected by decimetric-scale subsidence will require a detailed studied specific species and large numbers of measurements for acceptable accuracy [39, 69].

## Supporting information

S1 Table Geographic locations of the sites where benchmarks were measured, mobile GPS stations were installed and geodesic data from continuous GPS stations located nearby our study sites were collected. (DOCX)

S1 File Data of benchmark heights and upper level of biota. (XLSX)
