# Supplementary material for: Calibrating coseismic coastal land-level changes during the 2014 Iquique (Mw=8.2) earthquake (northern Chile) with leveling, GPS and intertidal biota
Source: PLoS One. 2017 Mar 23;12(3):e0174348. doi: 10.1371/journal.pone.0174348 (PMC5363922; doi:10.1371/journal.pone.0174348)
Supplement: S1 Table — (DOCX) [file pone.0174348.s001.docx]

**SUPPLEMENTARY MATERIAL**

**S1 Table**. Geographic locations of the sites where benchmarks were measured, mobile GPS stations were installed and geodesic data from continuous GPS stations located nearby our study sites were collected.

| **sites** | **benchmarks** | **mobile GPS** | **continuous GPS** | **latitude** | **longitude** |
| --- | --- | --- | --- | --- | --- |
|  |  |  |  |  |  |
| **IACR, *ca.* Corazones** |  |  | **X** | **18°28'49.45"S** | **70°19'55.33"W** |
| **Corazones** | **X** |  |  | **18°31'44.30"S** | **70°19'21.60"W** |
| **PSGA, Pisagua** |  |  | **X** | **19°35'50.56"S** | **70° 7'22.84"W** |
| **Pisagua norte** | **X** | **X** |  | **19°35'19.10"S** | **70°12'21.80"W** |
| **Pisagua sur** | **X** | **X** |  | **19°35'37.50"S** | **70°12'35.40"W** |
| **IQQE, *ca.* Cavancha** |  |  | **X** | **20°16'24.75"S** | **70° 7'54.17"W** |
| **Cavancha** | **X** | **X** |  | **20°14'10.39"S** | **70° 9'10.00"W** |
| **Quintero** | **X** | **X** |  | **20°36'26.20"S** | **70°11'51.70"W** |
| **AEDA, *ca.* Quintero** |  |  | **X** | **20°32'46.36"S** | **70°10'39.55"W** |
| **Chanavayita** |  | **X** |  | **20°42'7.50"S** | **70°11'23.20"W** |
| **CRSC, *ca.* Chanavaya** |  |  | **X** | **20°55'3.63"S** | **70° 4'47.21"W** |
| **Chanavaya** | **X** |  |  | **20°53'13.00"S** | **70° 8'16.60"W** |
|  |  |  |  |  |  |
